# Semaglutide ameliorates diabetes-associated cognitive dysfunction in mouse model of type 2 diabetes

**DOI:** 10.1371/journal.pone.0326897

**Published:** 2025-07-03

**Authors:** Yan Zhu, Yi He, Hongyan Yang, Yanbo Gao, Yan Wang, Peiqing Liu, Mengjuan Zhang

**Affiliations:** Department of Endocrinology, Baotou Central Hospital, Baotou, China; Zhejiang University of Technology, CHINA

## Abstract

**Background:**

Type 2 diabetes mellitus (T2DM) is associated with cognitive dysfunction, which significantly impacts the quality of life. Semaglutide, a glucagon-like peptide-1 (GLP-1) receptor agonist, has shown potential neuroprotective effects. This study investigates the efficacy of semaglutide in ameliorating cognitive dysfunction in a mouse model of T2DM.

**Methods:**

Male C57BL/6J mice were fed a high-fat diet for four weeks and received a single intraperitoneal injection of streptozotocin (150 mg/kg) to induce T2DM. All mice were divided into four groups: control, diabetes control (T2DM), semaglutide treatment (semaglutide, 0.1 mg/kg) and dapagliflozin treatment (dapagliflozin 1 mg/kg). Cognitive function was assessed using the Morris water maze (MWM) test. Histomorphological analysis of hippocampal tissues was performed using H&E and Nissl staining. Immunofluorescence was used to assess LRP1 expression and apoptosis. Biochemical analyses measured oxidative stress markers (SOD, MDA) and inflammatory cytokines (IL-1β, IL-6, TNF-α, CRP).

**Results:**

Semaglutide treatment significantly reduced blood glucose levels in diabetic mice. In the MWM test, semaglutide-treated mice showed reduced escape latencies, indicating improved spatial learning and memory. Histomorphological analysis revealed preserved neuronal structure in the hippocampus with reduced neuronal damage and apoptosis in the semaglutide-treated group. Immunofluorescence showed increased LRP1 expression and decreased apoptosis. Biochemical analyses indicated that semaglutide reduced oxidative stress and inflammatory markers, further supporting its neuroprotective effects.

**Conclusions:**

Semaglutide effectively ameliorates cognitive dysfunction in T2DM mice, likely through mechanisms involving the reduction of oxidative stress, inflammation, and neuronal apoptosis. These findings suggest that semaglutide has potential as a therapeutic agent for managing diabetes-associated cognitive decline. Further research, including long-term studies and clinical trials, is necessary to validate these findings and explore the broader applicability of semaglutide in treating cognitive impairments in diabetic patients.

## Introduction

Type 2 diabetes mellitus (T2DM) is a chronic metabolic disorder marked by persistent hyperglycemia due to insulin resistance and insufficient insulin secretion. The global prevalence of T2DM has been increasing [1], presenting significant public health challenges because of its links to serious complications, including cardiovascular diseases, nephropathy, retinopathy, and neuropathy [2]. Among these complications, cognitive dysfunction has emerged as a critical concern, significantly impacting the quality of life and daily activities of individuals with diabetes [3,4]. This diabetes-associated cognitive decline encompasses a range of deficits, including impairments in memory, executive function, and processing speed, which in turn elevate the risk of developing dementia [5,6].

Glucagon-like peptide-1 (GLP-1) is a 30-amino acid endogenous incretin hormone that enhances insulin secretion in a glucose-dependent manner, suppresses glucagon release, and delays gastric emptying, thereby contributing to glycemic regulation [7]. GLP-1 plays a vital physiological role through its interaction with GLP-1 receptors [8]. Recent studies have explored the potential of GLP-1 receptor agonists (GLP-1 RAs) as therapeutic agents for managing T2DM and its associated cognitive deficits [9,10]. Beyond their metabolic effects, GLP-1 and its analogs have shown neuroprotective properties, including the promotion of neurogenesis, reduction of oxidative stress, and suppression of neuroinflammation [11–14]. Semaglutide, a long-acting GLP-1 receptor agonist, has shown promise in preclinical studies for enhancing cognitive function in animal models, positioning it as a potential candidate for treating diabetes-related cognitive decline [15].

Cognitive dysfunction is increasingly recognized as a significant complication of Type 2 diabetes mellitus (T2DM), with patients experiencing impairments in memory, executive function, and learning abilities [16,17]. This diabetes-associated cognitive decline not only reduces quality of life but also complicates disease management and increases healthcare burden [18]. Despite the growing prevalence of this complication, treatment options specifically targeting cognitive dysfunction in diabetes remain limited [19].

This study aims to investigate whether semaglutide, a glucagon-like peptide-1 (GLP-1) receptor agonist approved for T2DM treatment, can effectively alleviate cognitive dysfunction in diabetic mouse models. GLP-1 receptor agonists have emerged as promising candidates for addressing neurological complications, as GLP-1 receptors are expressed throughout the brain, particularly in regions critical for cognitive function [20,21]. Previous research has demonstrated that GLP-1 receptor activation may improve cognitive outcomes by enhancing synaptic plasticity, reducing neuroinflammation, and protecting against neuronal apoptosis [22,23].

To distinguish between cognitive benefits derived from improved glycemic control versus direct neuroprotective mechanisms, we employ a comparative approach by including dapagliflozin, a sodium-glucose cotransporter-2 (SGLT2) inhibitor, as a reference treatment. This comparison is methodologically crucial because both medications effectively lower blood glucose levels but operate through fundamentally different mechanisms. Dapagliflozin primarily acts in the kidneys to increase urinary glucose excretion, addressing peripheral aspects of diabetes without directly targeting central nervous system pathways [24,25]. In contrast, semaglutide not only improves glycemic control but potentially exerts direct effects on brain function through GLP-1 receptors expressed in key cognitive regions, including the hippocampus [26].

In this investigation, we utilize the Morris water maze (MWM) test—a well-validated paradigm for assessing spatial learning and memory in rodent models—to evaluate cognitive function in diabetic mice following treatment with either semaglutide or dapagliflozin. Additionally, we examine biochemical markers of neuroinflammation, synaptic plasticity, and insulin signaling in hippocampal tissue to elucidate potential mechanisms underlying cognitive changes.

By comparing these two antidiabetic agents with different mechanisms of action, we aim to delineate the extent to which cognitive improvements correlate with glycemic control versus specific neuroprotective effects mediated through GLP-1 receptor signaling. This approach may provide valuable insights into the unique therapeutic potential of semaglutide for addressing diabetes-associated cognitive dysfunction and inform future clinical applications for patients experiencing cognitive decline in the context of T2DM.

## Methods

### Reagents and animals

**Fig 1:**
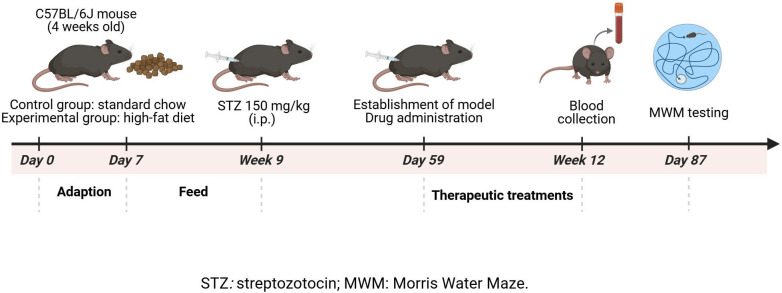
Schematic diagram of the study design.

Male C57BL/6J mice, aged 4 weeks and weighing approximately 18–20 g, were obtained from Beijing Vital River Laboratory Animal Technology. The animals were housed under controlled conditions, maintaining a temperature between 22°C and 25°C with a 12-hour light/dark cycle. Mice were divided into two groups: a control group fed standard chow and an experimental group provided with a high-fat diet. The high-fat diet consisted of 59% regular feed, 20% sucrose, 18% pork fat, and 3% egg yolk powder. This dietary regimen continued for 6–8 weeks until the mice reached a body weight of 30–35 g. All procedures received ethical approval from the Animal Ethics Committee of Baotou Central Hospital (Approval ID: KYLL2022−014). Surgical interventions were performed under sodium pentobarbital anesthesia, with careful measures taken to minimize discomfort and stress to the animals. At the end of the experiments, mice were sacrificed using CO2 asphyxiation in accordance with established protocols for humane euthanasia. Pain mitigation and distress prevention measures, including careful handling and monitoring, were employed during all experimental steps. The schematic diagram of the experimental design is shown in Fig 1.

### Establishment of diabetic mice

Following an overnight fast, mice were injected intraperitoneally with streptozotocin (STZ) at a dose of 150 mg/kg to induce T2DM [27], while control mice received an equivalent volume of sodium citrate buffer. Blood glucose levels were measured three days post-injection, with mice displaying glucose levels of ≥16.7 mmol/L considered diabetic. The diabetic mice were then randomly assigned to one of three groups: diabetes control (T2DM), semaglutide treatment (0.1 mg/kg, i.p., administered every other day) [28], and dapagliflozin treatment (1 mg/kg, p.o., administered daily) [25]. Semaglutide was administered for a period of 30 consecutive days.

### Morris water maze (MWM)

Cognitive function was assessed using the Morris water maze (MWM) test to evaluate spatial learning and memory. The apparatus consisted of a black cylindrical water tank (120 cm in diameter, 50 cm in height) filled with water made opaque with non-toxic white paint and maintained at a temperature of 22–25°C. A circular platform (10 cm in diameter) was submerged 1 cm below the water surface in the fourth quadrant of the tank. The testing protocol consisted of a 4-day acquisition phase followed by a probe test on the fifth day.

During the acquisition phase (days 1–4), each mouse performed four trials daily with a 15-minute inter-trial interval. For each trial, mice were placed into the water at one of four designated starting positions (north, east, south, or west), facing the tank wall. Animals were allowed to swim for a maximum of 60 seconds to locate the hidden platform. Mice that successfully found the platform were permitted to remain on it for 15 seconds before being returned to their home cage. Those unable to locate the platform within the allotted time were gently guided to it and allowed to remain there for 15 seconds.

Performance was recorded and analyzed using ANY-maze video tracking software (version 6.0, Stoelting Co., Wood Dale, IL, USA). A digital camera (30 frames per second) mounted directly above the center of the maze captured each trial. The software tracked the animal’s position in real-time by detecting the contrast between the mouse’s dark coat color and the white opaque water. During the acquisition phase, we measured escape latency (seconds to reach the platform), path length (total distance traveled in centimeters), and average swimming speed (cm/s) for each trial.

The probe trial was conducted 24 hours after the final acquisition session (day 5). For this test, the platform was removed from the tank, and mice were allowed to swim freely for 60 seconds, starting from a position opposite to the target quadrant. During this probe trial, we recorded the percentage of time spent in the target quadrant (where the platform was previously located), the number of platform location crossings (frequency of swimming over the former platform location), and the latency to first platform crossing. Heat maps representing swimming paths were generated for each animal to visualize search strategies.

All data were automatically recorded by the software and subsequently verified by an experimenter blind to the treatment groups to ensure accuracy and prevent bias. To minimize potential confounding effects of circadian rhythm on performance, all testing was conducted during the same time window each day (between 9:00 AM and 12:00 PM).

### Histomorphological analysis

At the end of the treatment period, the mice were sacrificed, and their brains were rapidly extracted. Hippocampal tissues were fixed in 10% formaldehyde, embedded in paraffin, and sliced into 4 μm sections for hematoxylin and eosin (H&E) staining as well as Nissl staining. Neuronal morphology within the hippocampus was then examined under a light microscope at 400x magnification.

### Immunofluorescence

Immunofluorescence analysis was performed to assess the expression of LRP1 and apoptosis within hippocampal tissues. Tissue sections were first incubated with primary antibodies against LRP1 (Rabbit, Abclonal, A14439, diluted at 1:200) and the neuronal marker NeuN (Rabbit, Affinity, DF6145, diluted at 1:200), followed by incubation with appropriate secondary antibodies: Goat Anti-Rabbit IgG Secondary Antibody (HRP, sino biological, SSA004, diluted at 1:1000) and Anti-rabbit IgG (H + L), F(ab’)2 Fragment (Alexa Fluor® 555 Conjugate, CST, #44413, diluted at 1:1000). Apoptotic cells were detected using the TUNEL assay. Fluorescent images were acquired using a fluorescence microscope, and data were quantified with ImageJ software.

### Biochemical analysis

Biochemical analyses included measurements of blood glucose, serum insulin and inflammatory markers (IL-1β, IL-6, TNF-α, and CRP). Blood samples were collected from the abdominal aorta under anesthesia, with serum separated by centrifugation and stored at −80°C for later analysis. Blood glucose levels were determined using a glucose meter. Levels of malondialdehyde (MDA) were assessed using the Malondialdehyde (MDA) Assay Kit (Jiangsu Aidisheng Biological Technology Co., Ltd., ADS-W-YH002, 100T/96S), and superoxide dismutase (SOD) levels were measured with the Total SOD Activity Assay Kit (WST-8 method) (Jiangsu Aidisheng Biological Technology Co., Ltd., ADS-W-KY011, 100T/96S). Inflammatory markers were measured using specific ELISA kits: Mouse Interleukin 1 Beta (IL-1β) ELISA Kit (Shanghai Jonlnbio Industrial Co., Ltd., JL18442, 48T), Mouse Interleukin 6 (IL-6) ELISA Kit (Shanghai Jonlnbio Industrial Co., Ltd., JL20268, 48T), Mouse Tumor Necrosis Factor Alpha (TNFa) ELISA Kit (Shanghai Jonlnbio Industrial Co., Ltd., JL10484, 48T), and Mouse C Reactive Protein (CRP) ELISA Kit (Shanghai Jonlnbio Industrial Co., Ltd., JL13196, 48T).

### Data analysis

All data are expressed as mean ± standard deviation (SD). Statistical analysis was conducted using GraphPad Prism 9.0 software. Group differences were evaluated using one-way ANOVA, followed by Tukey’s post-hoc test for pairwise comparisons. A *p*-value of <0.05 was considered indicative of statistical significance.

## Results

### Effects of semaglutide treatment on blood glucose levels of T2DM mice

**Fig 2:**
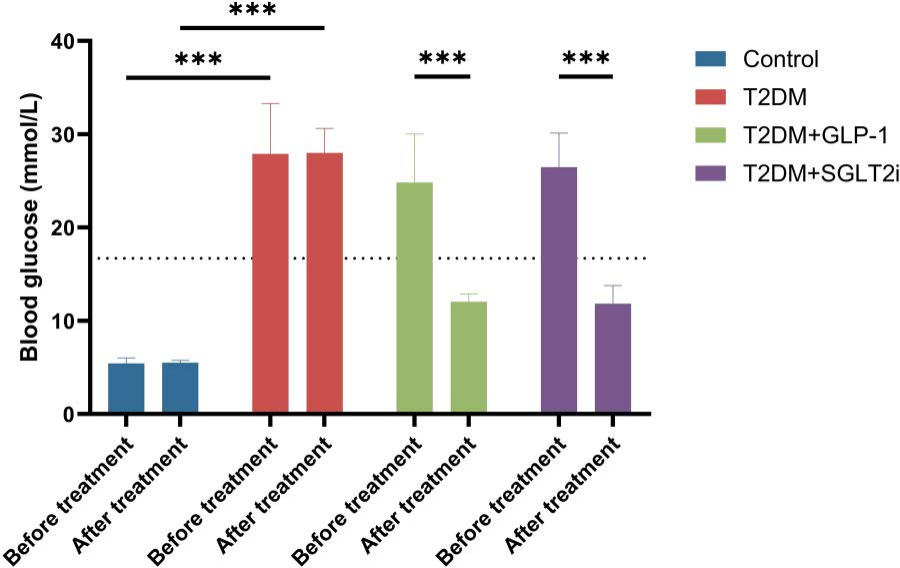
Effects of Semaglutide and Dapagliflozin on Blood Glucose Levels in T2DM Mice. Blood glucose levels were measured before and after 4 weeks of treatment across four experimental groups: Control (blue), T2DM (red), T2DM+GLP-1 (green, semaglutide-treated), and T2DM+SGLT2i (purple, dapagliflozin-treated). Before treatment, all diabetic groups (T2DM, T2DM+GLP-1, and T2DM+SGLT2i) showed significantly elevated blood glucose levels compared to the Control group (***p < 0.001). After treatment, the Control group maintained normal glycemic levels, while the untreated T2DM group remained hyperglycemic. Both semaglutide and dapagliflozin treatments resulted in significant reductions in blood glucose levels compared to pre-treatment values and the untreated T2DM group (***p < 0.001). Data are presented as mean ± standard deviation (SD).

Fig 2 illustrates the blood glucose levels (mmol/L) in different groups of mice before and after treatment. Prior to treatment, all diabetic groups (T2DM, T2DM+GLP-1, and T2DM+SGLT2i) showed significantly elevated blood glucose levels compared to the Control group (*p* < 0.001). Following treatment, blood glucose levels in the control group remained stable and within the normal range. The untreated T2DM group showed no notable change, retaining significantly higher glucose levels than all other groups (*p* < 0.001).

In contrast, both the semaglutide (T2DM+GLP-1) and dapagliflozin (T2DM+SGLT2i) groups experienced marked reductions in blood glucose levels compared to their pre-treatment values and the untreated T2DM group (*p* < 0.001). The semaglutide-treated group demonstrated a more pronounced glucose-lowering effect, with levels approaching the diagnostic threshold for diabetes (16.7 mmol/L, indicated by the dotted line in the figure). While both treatments significantly improved glycemic control, neither completely normalized blood glucose to control levels. These results suggest that both semaglutide and dapagliflozin treatments effectively lower blood glucose levels in T2DM mice, with semaglutide showing a slightly more robust effect.

### Effects of semaglutide treatment on metabolic parameters of T2DM mice

The baseline characteristics table (Table 1) summarizes key findings regarding the levels of various markers across the different groups. A comparison of the groups reveals that T2DM mice consistently have elevated levels of ALT, AST, TG, CHO, and FFA, along with reduced levels of HDL and LDL, compared to the control group. Interestingly, mice treated with either semaglutide or dapagliflozin display intermediate values for these markers, suggesting a potential impact of these treatments on metabolic parameters. These findings emphasize the distinct metabolic profiles of T2DM mice and suggest possible variations in response to different antidiabetic therapies.

**Table 1:** Metabolic characteristics of T2DM mice.

	Control(n = 3)	T2DM(n = 3)	Semaglutide(n = 3)	Dapagliflozin(n = 3)
**ALT(U/L)**	31.96 ± 5.408^**^	80.31 ± 7.383	51.41 ± 8.696^**^	51.64 ± 7.870^**^
**AST(U/L)**	159.72 ± 42.368^***^	286.51 ± 14.244	179.31 ± 26.507^**^	180.17 ± 31.709^**^
**TG(mmol/L)**	0.55 ± 0.146^**^	1.20 ± 0.270	0.67 ± 0.101^*^	0.67 ± 0.267^*^
**CHO(mmol/L)**	2.13 ± 0.328^***^	4.91 ± 0.711	3.16 ± 0.280^**^	3.20 ± 0.651^**^
**HDL(mmol/L)**	1.04 ± 0.172^**^	0.42 ± 0.105	0.65 ± 0.066	0.65 ± 0.246
**LDL(mmol/L)**	0.16 ± 0.061^**^	0.44 ± 0.044	0.29 ± 0.070^*^	0.30 ± 0.108
**FFA(mmol/L)**	0.17 ± 0.021^***^	0.49 ± 0.020	0.33 ± 0.025^***^	0.32 ± 0.026^***^

ALT, alanine aminotransferase; AST, aspartate aminotransferase; TG, triacylglycerol; CHO, total cholesterol; HDL, high-density lipoprotein; LDL, low-density lipoprotein; FFA, free-fatty acids.

Data are mean ± SD. ^*^P < 0.05 versus T2DM group,^**^P < 0.01 versus T2DM group, ^***^P < 0.001 versus T2DM group.

### Semaglutide ameliorates the learning and memory impairments in T2DM mice

**Fig 3:**
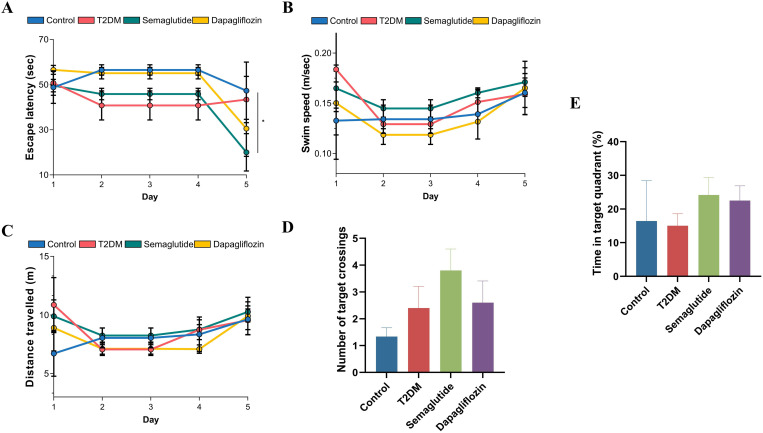
Assessment of cognitive function using the Morris water maze test. (A) Escape latency (time to find the hidden platform) over five training days. The semaglutide group (green) showed progressively shorter escape latency compared to the T2DM group (red), with the most pronounced difference on day 5. The dapagliflozin group (yellow) showed improvement primarily on day 5. The control group (blue) maintained consistent performance throughout training. (B) Swim speed during the training days showed no significant differences between groups, indicating that motor function was not a confounding factor in cognitive assessment. (C) Distance traveled before finding the platform across training days, showing patterns similar to escape latency results, with the semaglutide group demonstrating improved performance compared to the untreated T2DM group. (D) Number of target crossings during the probe trial. The semaglutide group showed the highest number of platform location crossings, followed by the T2DM group, control group, and dapagliflozin group. (E) Time spent in the target quadrant during the probe trial. Both treatment groups (semaglutide and dapagliflozin) spent more time in the target quadrant compared to the control and T2DM groups, with the semaglutide group showing the highest percentage.Data are presented as mean ± standard deviation.Statistical significance is indicated as *p < 0.05.

We then assessed the impact of semaglutide and dapagliflozin on spatial learning and memory using the Morris Water Maze (MWM) test. As illustrated in Fig 3A, over the five days of training, the semaglutide group (green) showed progressively shorter escape latencies compared to the T2DM group (red), with the most pronounced difference on day 5. The dapagliflozin group (yellow) showed improvement primarily on day 5, while the control group (blue) maintained consistent performance throughout training. Importantly, there were no significant differences in swim speed among the groups on any training day (Fig 3B), confirming that motor function was not a confounding factor in cognitive assessment. The distance traveled results (Fig 3C) showed patterns similar to escape latency, with the semaglutide group demonstrating improved performance compared to the untreated T2DM group. In the probe trial, the number of target crossings (Fig 3D) showed that the semaglutide group had the highest number of platform location crossings, followed by the T2DM group, control group, and dapagliflozin group. For time spent in the target quadrant (Fig 2E), both treatment groups spent more time in the target quadrant compared to the control and T2DM groups, with the semaglutide group showing the highest percentage.

In summary, these results suggest that semaglutide treatment enhances spatial learning and memory in T2DM mice, as evidenced by improved performance in both the training phase and probe trial of the MWM test. Dapagliflozin also showed beneficial effects on cognitive function, particularly in the probe trial measurements, though with some differences in performance pattern compared to semaglutide.

### Effects of semaglutide treatment on the morphological changes in hippocampus tissues from T2DM mice

**Fig 4:**
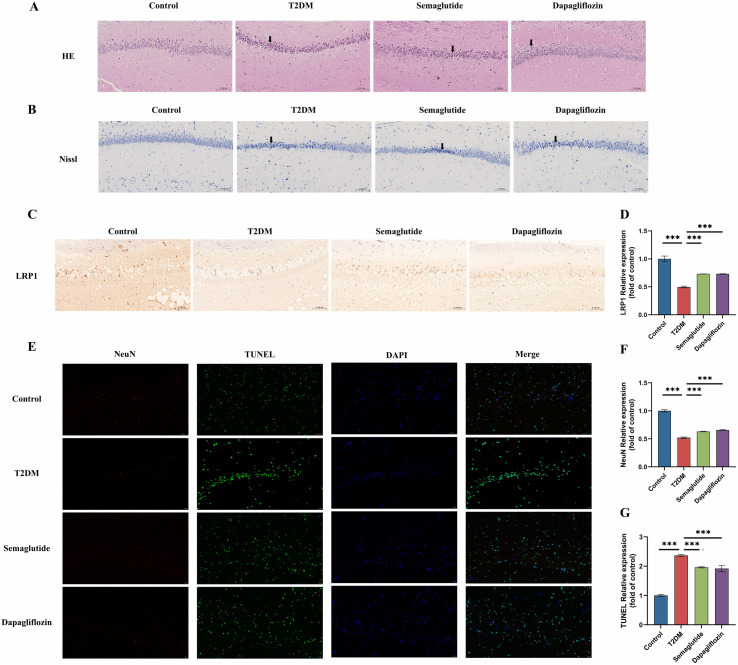
Effects of Semaglutide and Dapagliflozin on Neuronal Integrity, Apoptosis, and LRP1 Expression in the Hippocampal CA1 Region of T2DM Mice. (A) Hematoxylin and eosin (H&E) staining of hippocampal CA1 region shows preserved neuronal structure in the control, semaglutide and dapagliflozin groups compared to the T2DM group. Black arrows indicate nuclear pyknosis (condensed, shrunken nuclei) in the T2DM group, suggesting neuronal damage. (B) Nissl staining further confirms the preservation of neuronal integrity in control and treated groups, with the T2DM group showing reduced Nissl substance. Black arrows indicate areas of neuronal loss and structural disruption in the hippocampal CA1 region. (C) Immunohistochemical staining for LRP1 (Low-density lipoprotein receptor-related protein 1) reveals reduced expression in the T2DM group, with restoration observed in both semaglutide and dapagliflozin-treated groups. (D) Quantitative analysis of LRP1 expression shows significantly decreased levels in the T2DM group compared to control (***p < 0.001), while both semaglutide and da*p*agliflozin treatment significantly restored LRP1 expression compared to the T2DM group (***p < 0.001). (E) Double immunofluorescence staining for NeuN (neuronal marker, red) and TUNEL (a*p*optosis marker, green), with DAPI nuclear counterstain (blue). The merged images show substantially increased neuronal apoptosis in the T2DM group compared to control, with both semaglutide and dapagliflozin treatments reducing apoptotic signals. (F) Quantitative analysis of NeuN-positive cells demonstrates significant neuronal loss in the T2DM group compared to control (***p < 0.001), with both treatments *p*reserving neuronal populations compared to the untreated T2DM group (***p < 0.001). (G) Quantitative analysis of TUNEL-*p*ositive cells confirms significantly increased apoptosis in the T2DM group compared to control (***p < 0.001), with both semaglutide and da*p*agliflozin treatments significantly reducing neuronal apoptosis (***p < 0.001). Data are *p*resented as mean ± standard deviation (SD).

Neuronal damage in hippocampal tissues forms the pathological basis of cognitive dysfunction. In the current study, the morphological structure of hippocampal tissue was examined using H&E staining. As shown in Fig 4A and 4B, H&E staining (A) and Nissl staining (B) of hippocampal sections reveal the structural integrity of neurons. In the T2DM group, a significant reduction in neuronal density and increased cell damage were observed compared to the control group. Treatment with semaglutide and dapagliflozin appeared to preserve neuronal structure, with semaglutide showing a more pronounced effect in maintaining neuronal integrity. The hippocampal tissue from the T2DM group exhibited substantial disintegration of the pyramidal lamellar structure, along with fixed contraction of neuronal nuclei, increased extracellular space, and disorganization. Chronic treatment with semaglutide and dapagliflozin significantly reduced these abnormalities. Furthermore, in both the semaglutide- and dapagliflozin-treated groups, hippocampal neurons displayed better cell morphology, orderly arrangement, and uniform staining. In summary, these observations suggest that both semaglutide and dapagliflozin mitigate pathological degeneration in the hippocampus.

### Effect of semaglutide treatment on cell apoptosis in hippocampal tissues

Immunohistochemical staining for LRP1 (Fig 4C) showed decreased expression in the T2DM group compared to controls. Quantification of LRP1 levels (Fig 4D) confirmed this reduction (p < 0.001). Both semaglutide and dapagliflozin treatments significantly restored LRP1 expression. Additionally, double staining for NeuN (a neuronal marker) and TUNEL (an apoptosis marker) (Fig 4E) revealed a higher number of TUNEL-positive (apoptotic) cells in the T2DM group relative to controls, indicating increased apoptosis. Treatment with semaglutide significantly reduced the number of TUNEL-positive cells, suggesting a decrease in apoptosis (p < 0.001). Quantitative assessments of NeuN (Fig 4F) and TUNEL (Fig 4G) staining corroborated these observations, with significant improvements in both the semaglutide and dapagliflozin groups compared to the T2DM group, and semaglutide demonstrating a stronger effect (p < 0.001).

In summary, these findings indicate that semaglutide and dapagliflozin treatments help preserve hippocampal neuronal integrity, enhance LRP1 expression, and reduce apoptosis in diabetic mice, with semaglutide showing more pronounced protective effects.

### Effect of semaglutide treatment on the oxidative stress in hippocampal tissues

**Fig 5:**
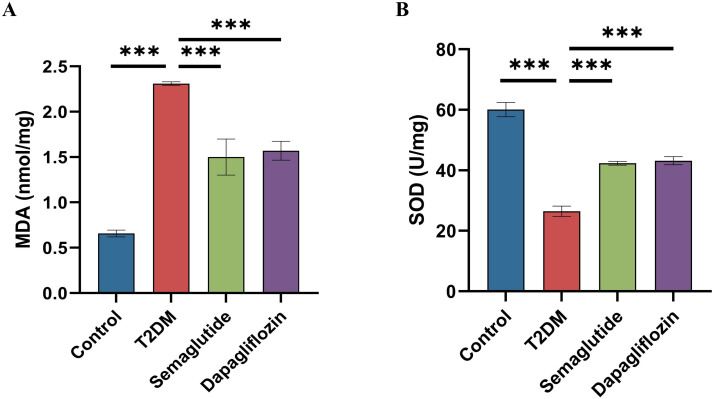
Effects of Semaglutide and Dapagliflozin on Oxidative Stress Markers in the Hippocampus of T2DM Mice. (A) MDA levels were significantly higher in the T2DM group compared to the control group (***p < 0.001). Both semaglutide and dapagliflozin treatments significantly reduced MDA levels compared to the T2DM group (***p < 0.001), with no significant difference between the treated groups. (B) SOD activity was significantly lower in the T2DM group compared to the control group (***p < 0.001). Treatment with semaglutide and dapagliflozin significantly increased SOD activity compared to the T2DM group (***p < 0.001), with no significant difference between the treated groups. Data are presented as mean ± standard deviation (SD). Statistical significance is indicated as ***p < 0.001.

Oxidative stress is closely associated with the pathology of cognitive dysfunction. As shown in Fig 5, both semaglutide and dapagliflozin treatments protect hippocampal tissues from oxidative stress, evidenced by increased activity of key antioxidant enzymes, such as SOD, and a reduction in neurotoxic lipid peroxidation end-product MDA levels. Additionally, these protective effects were more pronounced with semaglutide treatment, indicating that semaglutide may further mitigate oxidative stress in the hippocampal tissues of diabetic mice.

### Effects of semaglutide treatment on inflammation factor in T2DM mice

**Fig 6:**
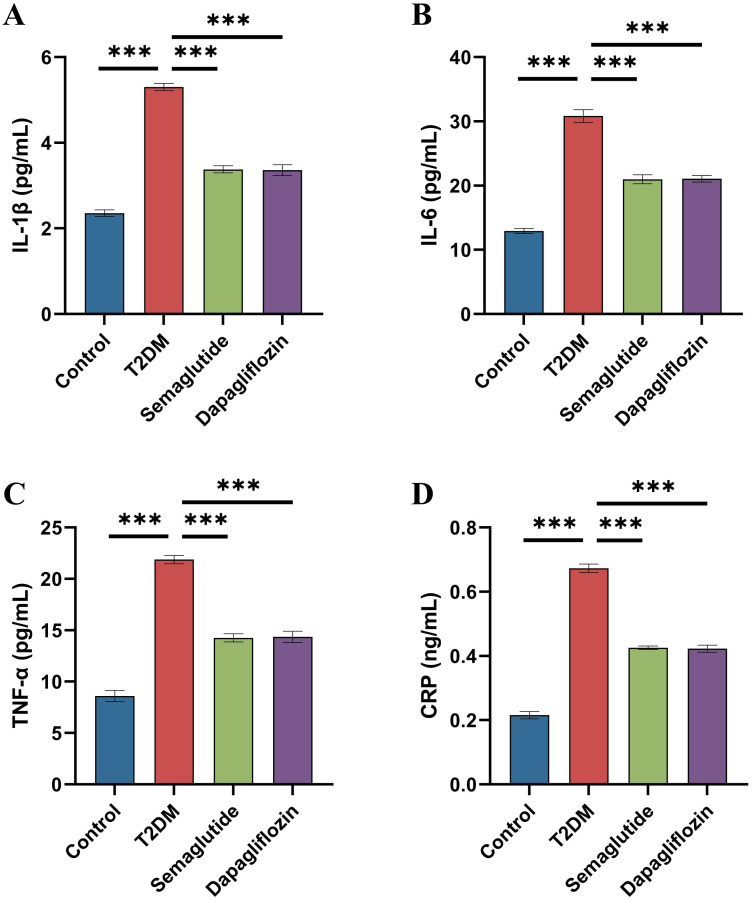
Effects of Semaglutide and Dapagliflozin on Inflammatory Markers in T2DM Mice. (A) IL-1β levels were significantly elevated in the T2DM group compared to the control group (***p < 0.001). Both semaglutide and dapagliflozin treatments significantly reduced IL-1β levels compared to the T2DM group (***p < 0.001), with no significant difference between the treated groups. (B) IL-6 levels followed a similar pattern, with significant reductions in the semaglutide and dapagliflozin groups compared to the T2DM group (***p < 0.001), and no significant difference between the two treatments. (C) TNF-α levels were also significantly higher in the T2DM group compared to the control group (***p < 0.001), with both treatments reducing TNF-α levels significantly (***p < 0.001), and no significant difference between the semaglutide and dapagliflozin groups. (D) CRP levels were elevated in the T2DM group, with significant reductions observed in the semaglutide and dapagliflozin groups compared to the T2DM group (***p < 0.001), and no significant difference between the treated groups. Data are presented as mean ± standard deviation (SD). Statistical significance is indicated as ***p < 0.001.

Fig 6 shows the serum levels of major inflammatory markers, including IL-1β, IL-6, TNF-α, and CRP, across the control, T2DM, semaglutide-treated, and dapagliflozin-treated groups. The T2DM group displayed significantly elevated levels of IL-1β, IL-6, TNF-α, and CRP compared to the control group (p < 0.001 for all markers). Both semaglutide and dapagliflozin treatments resulted in a substantial reduction in these inflammatory markers.

Specifically, IL-1β levels were significantly lower in both the semaglutide and dapagliflozin groups than in the T2DM group (p < 0.001), with semaglutide showing a slightly greater decrease. Similarly, IL-6 levels were significantly reduced in the semaglutide-treated group compared to the T2DM group (p < 0.001), while dapagliflozin also lowered IL-6 levels, though to a lesser extent (ns). TNF-α and CRP levels exhibited a similar pattern, with both treatments significantly decreasing their levels (p < 0.001), and semaglutide demonstrating a more pronounced effect overall. These findings suggest that semaglutide and dapagliflozin effectively reduce inflammation in diabetic rats, with semaglutide showing a stronger anti-inflammatory effect.

## Discussion

The current study reveals that semaglutide markedly improves cognitive impairment in type 2 diabetes mellitus (T2DM) mouse models. Our results indicate that semaglutide administration not only effectively lowers blood glucose levels but also enhances spatial learning and memory, demonstrated by shorter escape times in the Morris water maze (MWM) test. Moreover, histomorphological assessments showed that semaglutide maintained neuronal integrity in the hippocampus, mitigating neuronal injury and reducing apoptosis. Biochemical analyses further validate these observations, showing a reduction in oxidative stress and inflammatory markers in diabetic mice treated with semaglutide. Altogether, these findings imply that semaglutide provides neuroprotective benefits for cognitive function in T2DM mice, possibly through the attenuation of oxidative stress, inflammation, and neuronal apoptosis.

Our findings align with previous research highlighting the neuroprotective properties of GLP-1 receptor agonists in models of diabetes and neurodegenerative diseases. For instance, Jantrapirom et al. demonstrated that liraglutide, another GLP-1 receptor agonist, restored the phosphorylation states of IRS1, Akt, and GSK-3β, while also reducing beta-amyloid accumulation and tau hyperphosphorylation in a human neuroblastoma cell line [29]. Likewise, in vivo studies have shown that liraglutide helps prevent abnormalities in Akt and GSK-3β signaling and reduces tau phosphorylation associated with Alzheimer’s disease in the brains of diabetic mice [30,31]. Additionally, liraglutide has been shown to prevent the loss of brain insulin receptors in Alzheimer’s disease models [32]. These findings corroborate our results, suggesting that activation of GLP-1 receptors can alleviate cognitive impairments.

Oxidative stress occurs when there is an imbalance between pro-oxidant and antioxidant activities in the body, favoring oxidation. This imbalance can lead to the infiltration of inflammatory neutrophils, elevated protease secretion, and the generation of numerous oxidative intermediates [33]. Mitochondria, essential for aerobic metabolism and a primary source of reactive oxygen species (ROS) within cells, play a critical role in oxidative stress regulation [34]. In the nervous system, the modulation of mitochondrial function and oxidative stress by GLP-1 receptor agonists (RA) contributes to the alleviation of neurodegenerative conditions associated with diabetes [35]. Specifically, in diabetes-related Alzheimer’s disease (AD), GLP-1 enhances mitochondrial biogenesis and the antioxidant defense system by modulating the PGC-1α signaling pathway, directly mitigating tau hyperphosphorylation [36]. Despite these insights, the precise mechanisms through which GLP-1 RA influences mitochondrial function and oxidative stress remain incompletely understood. It is proposed that GLP-1 signaling may enhance mitochondrial biogenesis through the PGC-1α/nuclear respiratory factor-1/mitochondrial transcription factor A pathway, regulated by adiponectin/AMP-activated protein kinase (AMPK), and increase the expression of NAD-dependent protein deacetylase sirtuin 1 (SIRT1), which upregulates Parkin expression, thereby promoting mitophagy [37]. Additionally, evidence indicates that GLP-1 enhances endoplasmic reticulum (ER)-mitochondria interactions, leading to increased mitochondrial activity [38].

GLP-1 receptor agonists (RAs) have demonstrated anti-inflammatory properties in the central nervous system (CNS). In vitro studies under inflammatory conditions have shown that GLP-1 reduces the secretion of TNF-α-related cytokines and chemokines in BV-2 microglia [23]. Additionally, liraglutide has been found to lower the levels of activated microglia and astrocytes in the brain of mice experiencing chronic inflammation [39]. Moreover, treatment with liraglutide counteracted neuroinflammation by promoting the release of anti-inflammatory factors such as IL-10, TGF-β, and arginase 1(Bewick et al. [Unpublished]). In models of lipopolysaccharide (LPS)-induced inflammation, liraglutide suppressed the polarization of pro-inflammatory microglia while encouraging the shift towards an anti-inflammatory microglial state, reducing inflammatory cytokine levels and inhibiting the activation of the NF-κB pathway [40].

Our assessment of cognitive function relied on the Morris water maze test, which is widely recognized as a robust measure of spatial learning and memory—cognitive domains that are particularly vulnerable in diabetes-associated cognitive dysfunction. The significant improvements in escape latency observed in semaglutide-treated mice align with findings from previous studies utilizing other GLP-1 receptor agonists. However, we acknowledge that cognitive function encompasses multiple domains beyond spatial learning and memory, including executive function, working memory, and recognition memory, which may not be fully captured by the MWM test alone. Future studies would benefit from incorporating additional behavioral paradigms such as the Novel Object Recognition Test to assess recognition memory, the Y-maze to evaluate working memory, or the Barnes maze as an alternative assessment of spatial learning. Such a multi-test approach would provide a more comprehensive evaluation of the potential cognitive benefits of semaglutide across different cognitive domains affected in diabetes.

Our study stands out by focusing specifically on semaglutide, a long-acting GLP-1 receptor agonist, and its unique impact on cognitive impairment associated with diabetes. Unlike earlier research that mainly concentrated on reducing amyloid plaques and inflammation, our study emphasizes the broader neuroprotective actions of semaglutide, particularly its effects on mitigating oxidative stress and apoptosis. This comprehensive perspective offers a more in-depth understanding of how semaglutide may improve cognitive function in T2DM.

A key strength of this study lies in its use of diverse methodological approaches to comprehensively assess the effects of semaglutide on cognitive function and neuroprotection. The Morris water maze (MWM) test yielded robust behavioral data, demonstrating improvements in spatial learning and memory [41]. Histological analyses, such as H&E and Nissl staining, provided valuable insights into the preservation of hippocampal neuronal structure. Furthermore, immunofluorescence targeting LRP1 and apoptosis markers enabled precise quantification of neuronal integrity and apoptosis [42]. Biochemical assays assessing oxidative stress markers (SOD, MDA) and inflammatory cytokines (IL-1β, IL-6, TNF-α, CRP) offered further insights into the mechanisms underlying semaglutide’s effects [43]. These integrative and thorough methods deliver a nuanced understanding of how semaglutide alleviates cognitive dysfunction, emphasizing its potential as a therapeutic option for addressing diabetes-related cognitive decline.

It is important to note that our study utilized exclusively male mice, which represents a limitation in the generalizability of our findings. This decision was made deliberately based on established sexual dimorphism in both diabetes progression and cognitive responses. Male C57BL/6J mice typically develop more severe hyperglycemia and insulin resistance when fed high-fat diets compared to females, allowing for a more pronounced diabetic phenotype within our experimental timeframe. Additionally, estrous cycle fluctuations in female mice can introduce hormonal variability that may influence cognitive performance and neuroinflammatory responses, potentially confounding the interpretation of drug effects. However, we acknowledge that this single-sex approach fails to capture potential sex-specific responses to semaglutide treatment. Furthermore, the prevalence and presentation of diabetes-associated cognitive impairment in humans show notable sex differences. Therefore, future research should prioritize parallel investigations in female mouse models to determine whether the cognitive benefits of semaglutide observed in our study extend to both sexes, and whether dosing requirements or mechanisms of action might differ between males and females. Such sex-inclusive approaches would better inform potential clinical applications of semaglutide for treating cognitive dysfunction in the diverse population of patients with type 2 diabetes.

While the findings of this study are promising, several limitations should be acknowledged and addressed in future research. Firstly, the study was limited to male mice, which may not adequately represent potential sex-based differences in the response to semaglutide treatment. Secondly, the eight-week duration of semaglutide administration may not capture its long-term impact on cognitive function and neuroprotection. Thirdly, although the study employed a wide range of biochemical and histological analyses, it did not delve into the specific molecular pathways involved in the neuroprotective effects of semaglutide. Finally, the direct applicability of these findings to humans remains uncertain without supporting evidence from clinical trials.

Future studies should consider several key improvements to expand our understanding of semaglutide’s effects on cognitive function in diabetes. First, investigations should include both male and female mice to explore potential sex differences in treatment response, as considerable sexual dimorphism exists in both diabetes progression and GLP-1 receptor signaling. Second, extending treatment periods beyond the eight-week duration used in our study would better assess the long-term effects of semaglutide on cognitive function and neuroprotection, providing insights into its sustained efficacy. Third, more detailed investigation of molecular pathways is needed to elucidate the specific mechanisms underlying semaglutide’s neuroprotective effects, particularly focusing on mitochondrial function, oxidative stress regulation, and neuroinflammatory pathways. Finally, clinical trials are essential to confirm the translational potential of these findings in human populations with T2DM, as animal models, while valuable, cannot fully recapitulate the complexity of human diabetes and cognitive decline. These methodological improvements will contribute to a more thorough understanding of semaglutide’s role in alleviating diabetes-related cognitive impairment and could ultimately facilitate its clinical application as a therapeutic strategy that addresses both metabolic and neurological aspects of T2DM.

These improvements will contribute to a more thorough understanding of semaglutide’s role in alleviating diabetes-related cognitive decline and could facilitate its clinical application.

## Conclusions

This study demonstrates semaglutide’s promising therapeutic potential for cognitive dysfunction in type 2 diabetes mellitus. Our findings reveal that beyond glycemic control, semaglutide provides significant neuroprotection through preservation of hippocampal neuronal structure, reduction of oxidative stress, and mitigation of neuroinflammation. These neuroprotective effects translated to measurable improvements in spatial learning and memory in our T2DM mouse model. Despite these encouraging results, our work has limitations including the use of only male mice and relatively short treatment duration. Future research should investigate long-term cognitive outcomes, sex-specific responses, underlying molecular mechanisms, and validate these findings through well-designed clinical trials. By addressing these knowledge gaps, semaglutide may emerge as an important therapeutic option for managing both metabolic dysfunction and cognitive decline in patients with T2DM.
